# Surgical trainee education in benign anorectal disease: a scoping review

**DOI:** 10.1016/j.sopen.2025.05.001

**Published:** 2025-05-20

**Authors:** Eleanor G.R. Watson, Tony Y. Zhang, Hwa Ian Ong, David M. Proud, Helen M. Mohan

**Affiliations:** a University of Melbourne, Parkville, VIC 3052, Australia; b Department of Surgery, Austin Health, 145 Studley Road, Heidelberg, VIC 3084, Australia

**Keywords:** General surgery, Education, Medical, Rectal diseases, Curriculum

## Abstract

**Background:**

Benign anorectal diseases such as haemorrhoids, perianal abscesses and fistulas are prevalent and disabling conditions that can be difficult to diagnose and treat.

This review aims to evaluate current education for training doctors around these diseases to inform the revision and development of surgical curricula.

**Materials and methods:**

A literature search was conducted in MEDLINE, Embase and Google Scholar and data from included articles were charted in a semi-structured table. Quantitative outcomes were presented using simple descriptive statistics. Qualitative data were analysed using a reflexive thematic analysis framework.

**Results:**

Ten studies were included. Most education was centred around haemorrhoids and delivered in the format of lectures and simulations. Harnessing the benefits of both on-demand and in-person content was key to optimising education delivery. In simulation studies, low-fidelity models were generally sufficient to meet educational objectives. There was universal agreement that the purpose of education was to supplement or prepare for clinical exposure, rather than to replace or ‘bridge gaps’ in experience. Education was found to be most useful and relevant when delivered to junior surgical or non-surgical cohorts.

**Conclusions:**

This review elucidates gaps in current literature on benign anorectal disease education and provides recommendations for the development and implementation of future education for surgical trainees. There is a need for education that addresses a broader range of anorectal conditions and has a greater focus on the retention and clinical translation of acquired knowledge and skills. Interventions should be designed to enhance clinical exposure and maintain relevance throughout training progression.

## Background

Benign anorectal diseases (BAD) such as haemorrhoids, anorectal abscesses and fistulas have a combined prevalence of 20–40 % in Western populations [[1], [2], [3], [4]]. Every year in Australia, over 100,000 procedures for BADs are performed, including more than 60,000 procedures for haemorrhoids [5]. Sequelae of BADs include chronic anorectal pain and bleeding, constipation, perianal sepsis and faecal incontinence [6]. It is crucial that general surgery trainees graduate with proficiency in the management of these prevalent and disabling conditions.

The diagnosis and management of BADs is associated with specific challenges. The symptoms can be difficult to differentiate, leading to misdiagnosis and improper management plans [7]. The narrow, angulated anorectal canal presents visual, spatial and ergonomic challenges, restricting surgical access to pathology and learning through observation [8,9]. Surgery for BADs is associated with significant complications including faecal incontinence and anal stenosis, as well as high rates of disease recurrence [6,[10], [11], [12]]. There is concern that graduates are not equipped to confidently manage the complexities of anorectal conditions at the commencement of consultant practice [[13], [14], [15], [16], [17], [18], [19], [20]].

Several studies have sought to characterise trainee deficiencies in the diagnosis and management of BADs. Diagnostic accuracy has been shown to be poor across all levels of training. [17,[21], [22], [23]] Published data on trainee caseload is generally lacking, but trainees in the United States and New Zealand are estimated to perform between 20 and 40 anorectal operations annually [[24], [25], [26]]. In the United Kingdom General Surgery curriculum, the number of cases considered sufficient for graduating trainees is 20 for fistula surgery and 15 for haemorrhoidectomy, with numbers for other anorectal operations not specified [27]. A study of newly-qualified surgeons in Italy found that none had operated on an adequate number of patients with anorectal disease by the end of their training [28]. In addition to suboptimal case numbers, the diversity and complexity of exposure appears to be lacking. Anorectal abscesses are more likely to be operated on by trainees, while surgeons are usually the primary proceduralists for procedures with greater risks of complications [24]. Many surgical colleges now mandate operative logbooks, but the degree to which case numbers correlate with operative proficiency remains contentious, particularly given considerable variation in rates of trainee skill acquisition [29,30]. Inadequate exposure to anorectal conditions in general surgical training has been attributed to the increasing subspecialisation of training; shifting of procedures to outpatient clinics; absence of specialist colorectal surgeons from training centres; and underrepresentation of anorectal disease in curricula and examinations [15,[31], [32], [33], [34]].

It is important that general surgical training curricula are continually reviewed to meet shortfalls in trainee proficiency and adapt to the changing workload and technology of the training environment. The Australian General Surgery Education and Training program has been re-extended in length on multiple occasions over concerns around trainee skill acquisition, most recently in 2022 [35,36]. In the United States, a national review of general surgery trainee logbooks found a dramatic decrease in operative experience since 1990 [37]. Many argue that relying on operative exposure is no longer sufficient to produce competent trainees [8,38,39]. These concerns received renewed attention in the COVID-19 pandemic, which saw operative exposure to BADs reduced through elective surgery cancellations [40]. Augmenting or standardising operative experience is difficult due to dependence on hospital turnover, willingness of supervising surgeons, and the number and experience of available trainees. The value of a formal curriculum to supplement clinical exposure is increasingly apparent, particularly as advances in simulation and virtual reality are providing opportunities for standardised skill acquisition outside of the operating theatre.

This scoping review aims to evaluate existing surgical education in BADs. Our objective is to describe key features of educational methods, examine the processes by which they were developed, and evaluate their efficacy, to inform the revision and development of proctology curricula in general surgical training. In providing a broad overview of the literature we also endeavour to identify gaps for further research.

## Material and methods

### Study design

A systematic scoping review was performed according to the five-stage approach of Arksey and O'Malley [41]: (1) identify the research question; (2) identify relevant studies; (3) study selection; (4) chart the data; and (5) collate, summarise and report the results. The reporting of this study was also guided by the PRISMA Extension for Scoping Reviews (PRISMA-ScR) [42]. As the first step in Kern's six-step approach to curriculum development in medical education [43], ‘Problem Identification and General Needs Assessment’, a scoping review was considered the most appropriate methodology for our overarching goal to develop and revise proctology curricula. No ethical approval or consent was required.

### Identifying the research question

Key concepts pertaining to the research aim were defined to align with the breadth of the scoping review approach. Education was defined as any method, intervention, strategy or technique for improving trainees' ability to diagnose or manage BADs. Benign anorectal diseases were any pathology relating to the anus and/or rectum, excluding neoplasia. Trainees were doctors or medical students who were not qualified surgeons and receiving education in the field of general surgery.

### Identifying relevant studies

A comprehensive search was performed to identify published and unpublished literature. Database searching was conducted using MEDLINE (Ovid) and Embase (Ovid) in November 2023 using key terms from the research aim, with no restrictions on publication date, article type or language (Supplementary Fig. 1). All literature identified in the search were uploaded to Covidence systematic review software (Veritas Health Innovation Ltd., Melbourne, Australia) for review. After duplicate removal, two reviewers (EW, TZ) independently screened article abstracts and then full texts against the eligibility criteria, with a third investigator (HM) available to resolve discrepancies in study selection. To identify relevant literature not returned through database searching, reviewers also searched Google Scholar and the reference lists of included articles. All article types were included except reviews, which had their reference lists searched for additional relevant literature.

### Study selection

Articles were eligible for inclusion if they described education for surgical trainees specifically relating to BADs. To avoid publication bias and enable gaps in the literature to be identified, articles were included irrespective of outcome reporting. Literature that listed BADs without any description or study of educational methodology (e.g. syllabuses) were excluded, as these were not thought to contribute a level of detail aligned with the aim of our review. Articles pertaining to laparoscopic, robotic or endoscopic surgical techniques were excluded as they require a discrete skillset and dedicated review. Articles focusing on paediatric surgery, urology, gastroenterology and obstetrics and gynaecology procedures were also excluded given their low relevance to general surgery. All studies selected for inclusion were stored in EndNote (Clarivate, Philadelphia, USA).

### Charting the data

To summarise baseline characteristics of included articles and provide a quantitative synthesis of results, data was extracted by authors EW and TZ into a semi-structured table, independently and then cross-referenced for consistency. The table evolved ad-hoc as salient components were identified through analysis of included texts.

### Collating, summarising, and reporting the results

Quantitative outcomes were presented using simple descriptive statistics. No statistical analysis was undertaken due to the heterogeneity of the data.

Analysis of qualitative data was guided by the reflexive thematic analysis (TA) phases described by Braun and Clarke [44]. In addition to reflexive TA being a common and accepted approach to qualitative analysis in scoping reviews [41,45], this allowed our analysis to be informed by the experiences, values, and assumptions of authors around trainee education in BADs. The authors, who are stakeholders from the positions of teacher and student, were thought to offer rich subjectivity facilitating a deductive approach.

Author EW (trainee general surgeon) completed dataset familiarisation and coding. Codes were clustered into patterns of meaning to develop initial themes, which were then mapped and reviewed against coded data to create a final set of themes. Together, EW, TZ (medical student) and HM (general and colorectal surgeon) reappraised themes for relevance against the entire dataset, with minor revisions to terminology.

In accordance with PRISMA-ScR guidelines, critical appraisal of individual sources of evidence was not undertaken as it is not a requirement for scoping reviews [42]. Simulator fidelity describes the sensory resemblance to reality. [46] This resemblance is contextual and a continuum, but to facilitate discussion and comparison of the simulators in this review, we delineated between high- and low-fidelity based on the framework proposed by Tun et al. [46] This differed from the reported fidelity in one study [47].

## Results

### Literature search

**Fig. 1:**
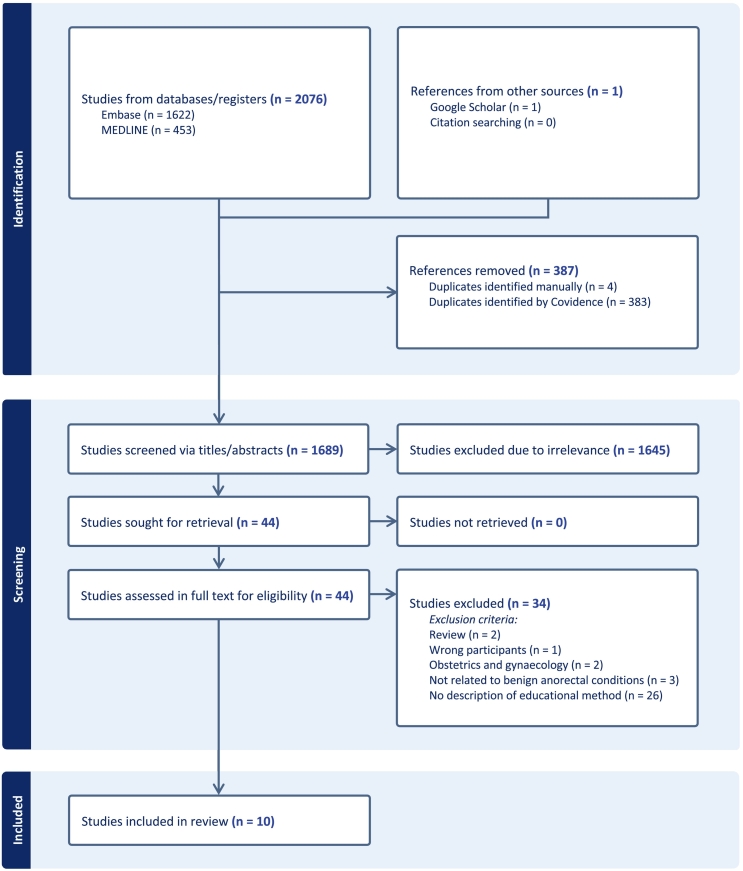
Literature search results.

The initial search identified 1689 unique studies, ten of which met the eligibility criteria and were included (Fig. 1). Studies were published from 2009 to 2022 and were conducted in the United States (*n* = 7), United Kingdom (*n* = 2), and Greece (*n* = 1). Two studies were randomised trials, five were quasi-experimental (pre/post, non-comparative design), two were cross-sectional analyses (post only, non-comparative design), and one was a quality improvement study. Two of these studies were conference abstracts with no associated full text. There were 660 participants across nine of the publications, who varied in seniority from medical students to surgeons and included doctors from specialties outside of general surgery. The additional publication described a website with 600–1300 monthly users. The characteristics of included texts are summarised in Table 1.

**Table 1:**
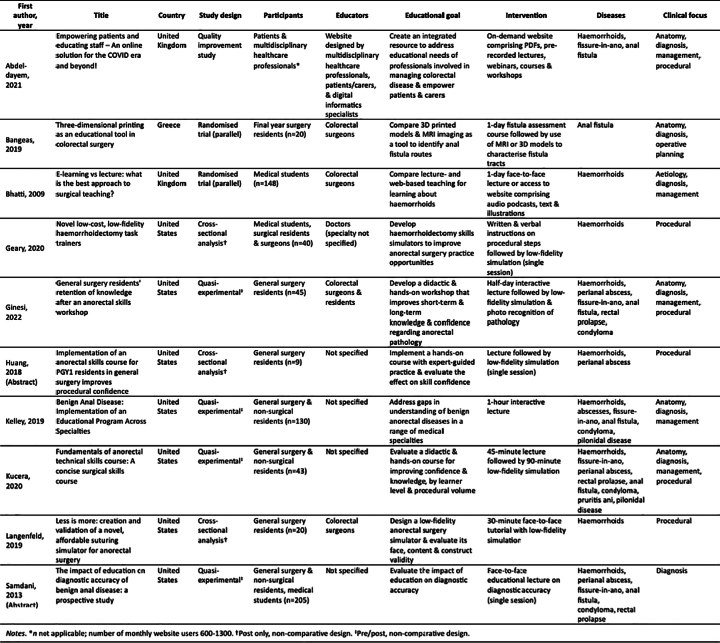
Summary of characteristics of included texts and their educational interventions.

### Features of education

The most common BAD featured in education was haemorrhoids, included in nine out of ten studies (Table 2). Studies of procedures focused on excisional haemorrhoidectomies [8,34,48,49], perianal abscess incision and drainage [34,47,49], rubber band ligation [34,47], and thrombosed haemorrhoid evacuation [47,49]. Education was delivered in the format of lectures [[50], [51], [52], [53]], simulation [48], or both [8,34,47,49,54]. Where both were used, lectures always preceded simulation. One study employed high-fidelity simulation, using three-dimensional printed models of fistula tracts [54]. All other studies used low-fidelity simulators, comprising a range of household objects and simple operative materials such as glass jars, toothpaste and drain tubing, which were used independently or to augment anatomical models and commercial task trainers. Additional educational strategies included podcasts, operative videos and interactive documents [50,53]. Half of publications specified the educators, who were usually colorectal surgeons. One study comprised online content only [50], while all other studies involved a face-to-face component. Of studies that reported education duration, all interventions were delivered over one day or less. Two studies using low-fidelity simulation reported the cost per simulator, which varied between $5–11 USD per unit. The three-dimensional printed models cost €3–5 per unit (excluding printer value).

**Table 2:** Benign anorectal diseases included in the General Surgeons Australia (GSA) technical curriculum [55] that were included by review articles.

	Review text									
GSAsyllabus	Abdel-dayem	Bangeas	Bhatti	Geary	Ginesi	Huang	Kelley	Kucera	Langenfeld	Samdani
Haemorrhoids										
Fissure-in-ano										
Anorectal abscess										
Anal fistula										
Ano-rectal incontinence										
Rectal prolapse										
Pruritus ani										
Proctitis										
Condyloma										

### Education development

Detail around intervention development was available for five publications. Three studies used a needs assessment to identify gaps in trainee understanding [[47], [48], [49]]. These were either conducted via informal discussions with students and colorectal faculty [48,49]; literature review [49]; or a formal written assessment [47], with education designed to focus on procedures or conditions where majority of respondents reported low confidence levels or provided poor management plans. The studies featuring websites were designed with input from colorectal faculty [50,53], while one sought additional representation from surgical trainees, patients, nurses, administrative staff and technology experts [50]. Published curriculum development frameworks were also used [49].

### Outcome measures

The outcome measures used by studies varied considerably and are summarised in Table 3.

**Table 3:** Outcome measures used by review texts.

	Review text									
Outcome measure	Abdel-dayem^a^	Bangeas^a^	Bhatti	Geary	Ginesi	Huang	Kelley	Kucera	Langenfeld	Samdani
Participant assessment at time of intervention										
Written examination (pre & post intervention)										
MCQ										
SAQ										
Spot diagnoses										
Self-rated confidence										
Practical examination (single timepoint)										
Time to complete task										
Quality of task completion										
No. of attempts at task										
Participant assessment post intervention (retention)^b^										
Written examination										
Self-rated confidence										
Intervention evaluation										
Free text feedback										
Rating scale										

a Texts used unique outcome measures: Abdel-dayem used website traffic to assess intervention efficacy; Bangeas used written examination post-intervention only and did not specify question format.

b Retention assessments were conducted six months post intervention. MCQ: Multiple choice questions. SAQ: Short answer questions.

Six interventions comprised a lecture [34,47,49,[51], [52], [53]], with half of these also including a simulation [34,47,49] or online [53] component. These studies measured participant knowledge before and after interventions using written examination. Examinations comprised multiple choice questions, short answer questions or spot diagnoses (images labelled with diagnoses that can made by visualising the common or classical presentation of disease). Likert scales were also used to assess self-perceived confidence in the diagnosis and management of BADs.

In all except one [51] of these studies, there was a statistically significant increase in knowledge and confidence-based scores following interventions. Bhatti et al. demonstrated improvement in knowledge around haemorrhoids with both face-to-face lectures and online audio-visual content, demonstrating a significant improvement in post-intervention test scores of 3–4 out of 25 points, and marginally better performance in participants using the online content [53]. Kucera et al. found a significant increase in confidence following a combined didactic and skills course, however this benefit was only observed in participants with less operative experience [34]. The exception was the study of Kelley et al., where following a 1 h lecture there was no significant difference in all participants' pre- and post- intervention test scores, and the scores of the general surgery cohort decreased (from 85 % correct on the pre-test to 79 % on the post-test).

Two studies comprised simulation only [8,48] and neither used written assessments, instead timing and rating the technical quality of tasks. Both studies used low-fidelity simulation to replicate the steps of an excisional haemorrhoidectomy. Novice and expert performance were compared at a single timepoint to validate task trainers. Experts performed tasks significantly faster than novices across studies. Langenfeld et al. showed that mean scores for knot-tying and suturing quality were significantly lower in novices versus experts, by approximately 2 points on a 5-point Likert scale [8]. Geary et al. demonstrated a significantly higher mean number of knot tie attempts in novices versus experts, but no difference in other indicators of procedural accuracy, such as gaps in wound closure and incomplete resection of pathological tissue [48].

Two studies were unique in their outcome assessments. Bangeas et al. studied the utility of MRI imaging versus three-dimensional printed models of patient anatomy for characterisation of fistula routes. Students completed a fistula course and used the models or images to describe fistula pathology via written examination, which was then assessed against the ‘correct’ pathology determined intraoperatively by surgeons. Students who used models scored consistently better on examinations than students using the MRI images. Changes in student performance with the intervention were not measured. The intervention of Abdel-dayum et al. comprised a website and measured online traffic as an indicator of efficacy [50]. The website had 600–1300 new monthly users, a low percentage of single interaction visitors (2.5 %), and a mean session duration use of 4 min and 20 s.

Seven studies included a method of program evaluation [8,[48], [49], [50], [51],^53^,^54^]. These comprised questionnaires with open-ended feedback fields or Likert scales measuring overall satisfaction and enjoyment and the usefulness, quality, efficiency and authenticity of the education. Users of educational websites rated the quality of the material and ease-of-use highly [50,53]. Overall, feedback from participants in simulations found these to be clearly instructed, relevant and useful. Free-text responses to evaluations have been addressed further in the thematic analysis.

Only two studies measured knowledge retention, both re-assessing students with written examinations six months post intervention. Ginesi et al., who implemented a combined lecture and hands-on skills course, found a small improvement in knowledge scores at six months versus immediately post intervention, while procedural confidence scores were unchanged [49]. In contrast, following the lecture-based session described by Kelley et al., there was a small reduction in knowledge on reassessment at six months [51].

### Thematic analysis

From thematic analysis of study methods and results, four themes were developed: Harnessing the benefits of on-demand and in-person content; The role of low-fidelity simulation; Defining the educational ‘scope of practice’; and Education for everyone, but stratified. Themes, subthemes and their associated codes have been mapped in Supplementary Fig. 2 and examples of coded text are shown in Supplementary Table 1.

### Harnessing the benefits of on-demand and in-person content

Studies reflected a favourable shift in surgical education from traditional textbook, paper-based formats to audio-visual material. Clinical examination was considered central to BAD diagnosis and operative planning, with spot diagnoses featured in many interventions. However, whether audio-visual material is best delivered in an on-demand or in-person format was contentious, with the benefits of each explored. On-demand education typically comprised websites, as well as ‘on-demand’ simulation models that students were able take home or recreate from household materials. Such formats were seen to be superior from the perspective of accessibility. They enabled trainees to revisit education on multiple occasions, favouring intervention durability and knowledge retention. The ability to choose when and where to access content minimised student time away from clinical activities and did not depend upon the availability of clinicians for delivery. Content could be revised opportunistically and in clinical contexts on smartphones, and delivered remotely at institutions where access to expertise was limited. Websites could be updated on a regular basis to maintain relevance and appropriate scope. In comparison, in-person lecture and simulation sessions were beneficial by enabling students to ask questions and receive real-time feedback. Such interventions also promoted student engagement and held students accountable for attendance, rather than relying on self-directed access to content. Authors endeavoured to harness the benefits of both formats, for example, one website offered a ‘live Q&A' chat to enable students to interact directly with content experts [50]. Although cost was not discussed as a major differentiating factor between on-demand and in-person formats, a low-cost intervention was prioritised by all studies, also supporting accessibility and reproducibility.

### The role of low-fidelity simulation

Five of the education interventions specifically featured anatomy, which was considered to be essential to understanding BADs. Anatomy education was most effective when accompanied by three-dimensional representations of structures and an interactive component, thus the predominance of simulation in this domain. Despite the acknowledged complexity of the anatomy, low-fidelity simulation was sufficient to replicate most challenges of operating in the anorectal canal, including visualising and differentiating between anorectal structures and operating within a confined space. Structures commonly prioritised were the rectal venous plexus and dentate line. Using transparent materials to create the anorectal canal optimised technical supervision and appreciation of the spatial relationships of structures. Although low-fidelity models were not accurate representations of anatomy, they provided authentic and useful procedural experience. The high-cost, resource-intensive nature of anatomically accurate simulators was seen as a significant disadvantage, and these were generally considered unnecessary to meet the educational objectives of studies.

### Defining the educational ‘scope of practice’

There was universal agreement that the purpose of interventions was to supplement, or prepare trainees for, clinical exposure. Education was not designed to replace clinical experience and the use of simulation to ‘bridge gaps’ in experience was generally not supported, despite the promising results of validation studies. Similarly, studies emphasised that simulation should not be used for formal assessment or grading of trainees. Instead, this should be performed within a clinical environment, at least until translation to practice can be established. Within studies of low-fidelity simulation, grading and assessment of students was performed for the purposes of intervention validation only. Studies asked trainees about the authenticity of models or their self-perceived procedural confidence, and the lack of clinical measures (e.g. intraoperative competence, patient outcomes) was a commonly cited limitation. High-fidelity models, such as three-dimensional printed models of fistula tracts, were mostly beneficial for characterising patient-specific anatomy and operative planning, fulfilling both an educational and clinical purpose.

### Education for everyone, but stratified

Studies identified the need for education to be delivered to doctors outside of the general surgical specialty given the prevalence of BADs. Nonetheless, where interventions were delivered to a broad audience, the importance of targeted education became apparent. Trainees with more surgical experience found education less useful and relevant, with the greatest benefits appreciated in junior general surgery cohorts or trainees from non-surgical specialties. Taught procedural skills were usually basic in nature, such as suturing or knot-tying. In novice cohorts, the role of simulation was seen to be skill familiarisation, versus skill-honing in experienced cohorts. In addition to having a multispecialty target audience, many interventions benefitted from multispecialty development. One avenue for achieving both targeted and broad education delivery was the use of ‘dynamic content’ in online formats, where different users have different experiences, rather than delivering the same intervention to all users irrespective of their characteristics (‘static content’). Abdel-dayum et al. broadened the delivery and development of their educational website to include patients, and offered parallel ‘portals’ for patient and medical cohorts [50].

## Discussion

This review presents the available data on education for BADs, an underrepresented domain of surgical training. Overall, studies supported the use of didactic and practical education interventions that were accessible, anatomy-focused, in an audio-visual format and delivered to junior surgical trainees. Interventions were frequently developed using needs assessments and were designed to complement or prepare for clinical experience. On-demand content was beneficial from the perspective of accessibility, while in-person content was preferable for student engagement and feedback. Based on our findings, we have devised key recommendations for the development and implementation of future BAD education (Table 4).

**Table 4:** Recommended features of future education for benign anorectal diseases (BADs).

Recommendation	Key finding
Audio-visual format	Examination is central to BAD diagnosis with spot diagnosis a prominent feature of education. The inclusion of visual content is essential, while audio enhances learner engagement.
Low-cost	Cost was universally prioritised. Expensive materials for high-fidelity simulators were unnecessary to achieve the objectives of BAD education. Minimising cost improves intervention accessibility and reproducibility.
Both on-demand and in-person content	On-demand content, delivered through websites or at-home simulators, was beneficial due to accessibility, while face-to-face education was superior from the perspective of feedback and learner engagement.
Reproducible and repeatable	Skill and knowledge retention were valued as highly as primary acquisition and were under-evaluated by studies. Interventions that could be reproduced or repeated in students' own time were thought to be most durable.
Updatable	The ability to update and sequentially expand educational content was seen as a significant advantage.
Anatomy focus	Understanding the anatomy of the anorectal canal is fundamental to understanding the diagnosis and management of BADs and was a focus of interventions.
Three-dimensional, interactive models	Three-dimensional representations of the anorectal canal that enabled students to interact with the anatomy facilitated understanding of the relationship between structures and associated pathology.
Target specific cohorts	Education should be stratified based on training field and level. Low-fidelity simulation is likely to be most beneficial for knowledge and skill acquisition in cohorts with less clinical and operative experience.
Beyond validation to transferability	Studies should move beyond traditional assessments of intervention validity to evaluating the transferability of acquired knowledge and skills to clinical contexts. This was a commonly cited limitation and area for further research.
Informed development	When used, formal and informal learning needs analyses were valuable in designing and validating interventions.
Clearly defined purpose	The role of educational interventions was to supplement or prepare students for clinical practice, not replace clinical exposure or assess clinical competence.

Low-fidelity simulation was a prominent feature of education interventions and was generally considered to be sufficient for educating junior trainees on basic procedures for BADs. A recent review of high- versus low-fidelity simulators in surgical training found that in majority of studies, both simulation types were equally as effective for improving performance compared to no training or didactic education alone [56]. High-fidelity simulation may actually be worse in novice cohorts due to overstimulation [57] and conferring overconfidence [58], and does not necessarily correlate with improved transfer of learning to clinical contexts [59]. Cadaveric and animal models are a form of high-fidelity simulation long used in surgical education, with clear value in understanding anatomy and learning to perform procedures on human tissues. However, as a limited resource, there is a role for high-fidelity haptic feedback devices, task trainers and computer-based simulators that provide anatomical detail and sensory realism to replicate these experiences. The role of high-fidelity simulation is likely better appreciated in the education of complex and high-stakes procedures, evidenced through recent developments in robotic surgery training [60,61].

General surgery training board curricula were not included in this review as they typically provide an overview of educational approaches for all surgical conditions, rather than specific methods for learning that aligned with our aim. Nonetheless, it useful to consider the available literature in the context of these curricula to identify educational opportunities. The published general surgery curricula of Australia and New Zealand and the United Kingdom are centred around clinical experience, defined competencies, and self-directed didactic learning [27,55]. Milestones and assessments commonly comprise operative logbooks, case discussions and supervised patient examinations. Aside from a small number of short courses and lists of suggested reading material, there are few references to specific methods of learning to complement clinical exposure. In comparison, the studies in this review described educational opportunities away from the bedside and operating theatre, and were focused on the process of achieving competency, rather than the competencies themselves. The proctology component of training board curricula may benefit from broader inclusion of didactic and simulation teaching methods external to the clinical environment. These are likely to exist to some degree in practice, as published curricula do not reflect the entirety of trainees' learning opportunities, which are largely governed by institutions. Nonetheless, formally integrating such strategies into curricula may promote uptake and development and assist in navigating the perpetual competition between trainee education and service requirements.

We identified a number of gaps in the available literature on BADs. Given the prevalence of these conditions and the volume of associated procedures, it is surprising that only ten publications on relevant education were available. Most publications focused on haemorrhoids, and there was a lack of interventions addressing other important conditions in the general surgery training syllabus (Table 2). The central dogma of anorectal surgery is to treat pathology while preserving continence, and the issue of sphincter injury, alongside other significant procedural complications such as anal stenosis and recurrence, were largely unaddressed in education, possibly because of the focus on novice learners. Our study population encompassed a breadth of specialties and experience to align with the scoping review approach, but further studies should establish the utility of interventions in specific trainee cohorts, given the stratified response to education. Although low-fidelity simulation was generally sufficient for learning, its flaws are likely to become pronounced in advanced trainees, and more research is required to develop education that remains relevant throughout training progression. Finally, surgical training varies considerably between countries and it would be useful to study education methods in populations outside of the United Kingdom and United States.

There were notable weaknesses with respect to the study designs of included texts.

A lack of formal intervention validation was apparent across studies, particularly those of simulations. Norman et al. defines five characteristics that should be evaluated for simulations: fidelity, reliability, validity, learning and feasibility [62]. All relevant studies specified simulation fidelity, although the delineation of high- and low-fidelity varied, a recognised issue in the medical simulation literature [56]. No studies formally assessed intervention reliability. All studies assessed construct or face validity through learner development, comparisons of novice and expert performance, or learner ratings of realism. Limited assessments of learning were undertaken, with studies only addressing two of Kirkpatrick's Four Levels of Training Evaluation [63]. Only two studies assessed knowledge retention [49,51]. Most studies addressed feasibility in some capacity, prioritising low-cost, reproducible interventions. Nonetheless, there should arguably be a shift from assessing intervention validity to the clinical transferability of learning, and further research should assess patient-centric outcomes of trainee knowledge and skill acquisition. A recently published guide to surgical simulation research highlights the ability for outcomes to be linked to real patients and surgeons as a major limitation [64]. The authors encourage study design to incorporate outcomes beyond attitudes and reactions to measure real-world behavioural change [64]. While any methodology may be used in surgical simulation research (so long as it is appropriate to answer the research question), an equally pertinent consideration is whether simulation is the correct approach for surgical teaching, learning or assessment in the first instance. Investigators should always consider whether they are studying a rare or risky event that makes education or research in a clinical environment inappropriate. [64]

This review has important limitations. Although we sought to identify published and unpublished literature, institutions frequently design and implement education without formally recording interventions and their outcomes. This review is likely to only present a portion of the BAD education strategies that are used in practice. Reporting bias is a pertinent issue in higher education research [65] and may have affected the available data, with only one study reporting a negative primary outcome [51]. Objective assessments of intervention efficacy were not possible in this review due to small sample sizes and heterogeneity across interventions, study design and outcome reporting. Although only ten publications were included, this review is an important initial step in generating discussion and ideas for the development and revision of proctology curricula.

## Conclusion

This review elucidates gaps in current literature on BAD education and provides recommendations for the development of targeted education interventions for surgical trainees. There is a need for education that addresses a broader range of anorectal conditions and has a greater focus on the retention and clinical translation of acquired knowledge and skills. Interventions should be designed to enhance clinical exposure and maintain relevance throughout training progression. As high-fidelity educational models become increasingly available, their role and efficacy will also need to be evaluated. Contributing to an evidence-based curriculum is important to standardise training quality and ultimately, optimise patient outcomes.

The following are the supplementary data related to this article.

Supplementary Fig. 1 Example of database search, conducted in MEDLINE (Ovid) in November 2023. Supplementary Fig. 1

Supplementary Fig. 2 Concept map of themes (dark blue), subthemes (light blue) and data codes (grey). Supplementary Fig. 2

Supplementary Table 1 A selection of code labels and collated data extracts. Supplementary Table 1

## CRediT authorship contribution statement

**Eleanor G.R. Watson:** Writing – review & editing, Writing – original draft, Visualization, Software, Methodology, Investigation, Funding acquisition, Formal analysis, Data curation, Conceptualization. **Tony Y. Zhang:** Writing – review & editing, Writing – original draft, Methodology, Investigation, Formal analysis, Data curation. **Hwa Ian Ong:** Writing – review & editing, Supervision. **David M. Proud:** Writing – review & editing, Supervision, Conceptualization. **Helen M. Mohan:** Writing – review & editing, Supervision, Formal analysis, Data curation, Conceptualization.

## Ethics approval

This study was a review. No ethics committee approval was required.

## Funding sources

This was an investigator-initiated study funded by a Research Training Program Scholarship provided to Dr. Eleanor Watson by the 10.13039/501100001782University of Melbourne.

## Declaration of competing interest

This review is being undertaken as part of Eleanor Watson's Doctor of Philosophy (University of Melbourne). The authors have no competing interests to declare.

## Data Availability

Data extracted from included studies and used in the analyses can be provided upon reasonable request to the corresponding author (EW).
