# Supplementary figures and images for: Surgical trainee education in benign anorectal disease: a scoping review
Source: Surg Open Sci. 2025 May 20;26:119–27. doi: 10.1016/j.sopen.2025.05.001 (PMC12167109; doi:10.1016/j.sopen.2025.05.001)

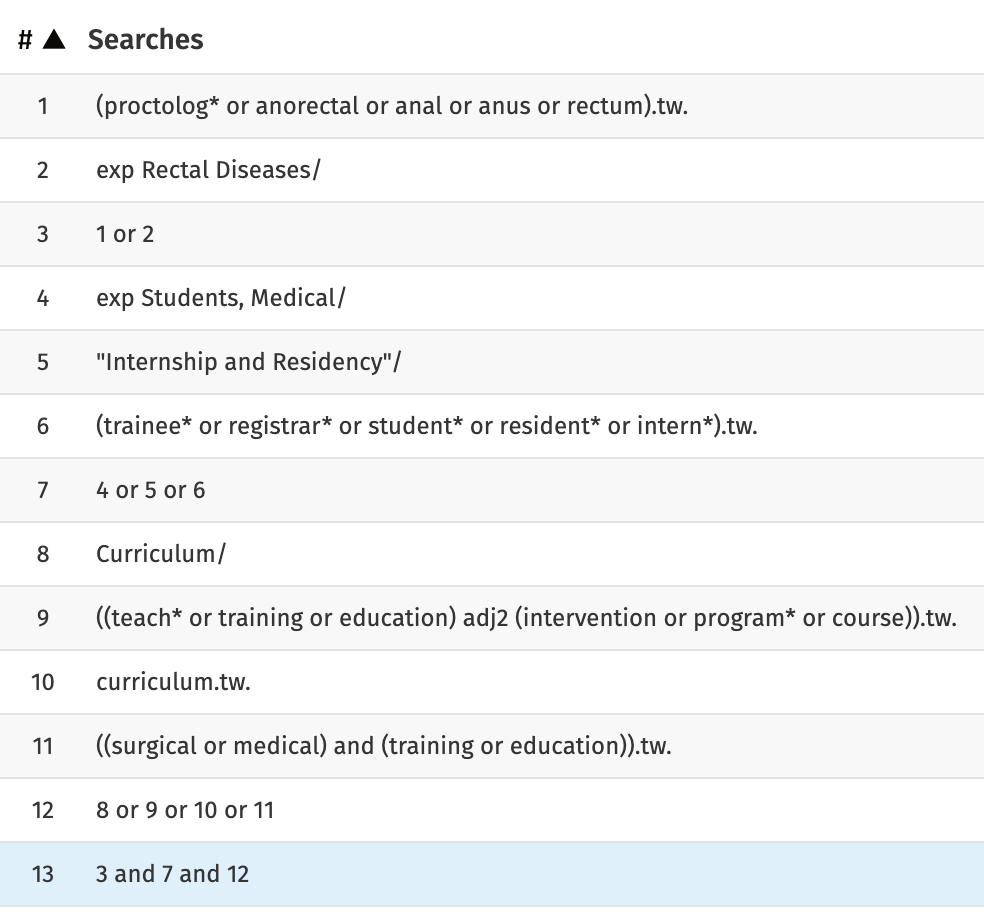
**Supplementary Figure 1.** Example of database search, conducted in MEDLINE (Ovid) in November 2023.

Supplement: Supplementary Fig. 1 — Example of database search, conducted in MEDLINE (Ovid) in November 2023. [file mmc1.docx]

**
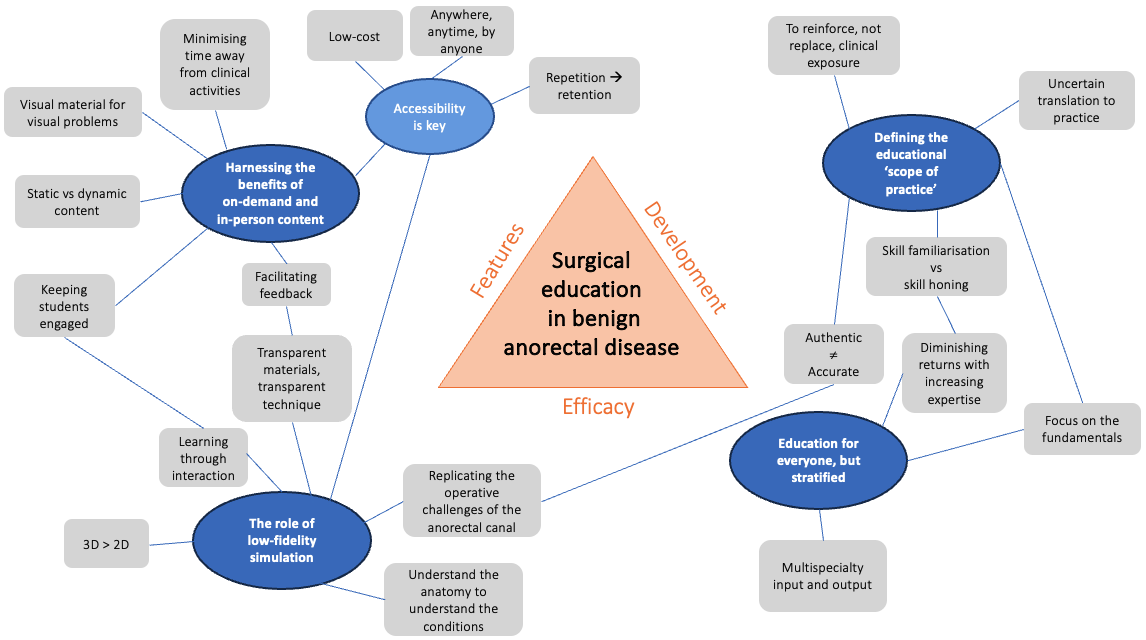
Supplementary Figure 2.** Concept map of themes (dark blue), subthemes (light blue) and data codes (grey).

Supplement: Supplementary Fig. 2 — Concept map of themes (dark blue), subthemes (light blue) and data codes (grey). [file mmc2.docx]
