# Supplementary material for: Surgical trainee education in benign anorectal disease: a scoping review
Source: Surg Open Sci. 2025 May 20;26:119–27. doi: 10.1016/j.sopen.2025.05.001 (PMC12167109; doi:10.1016/j.sopen.2025.05.001)
Supplement: Supplementary Table 1 — A selection of code labels and collated data extracts. [file mmc3.docx]

| **Code** | **Representative text** |
| --- | --- |
| Anywhere, anytime, by anyone | “The trainers [are] easy to use anywhere there is a flat surface” (Geary)  “The lightweight design and benchtop nature of the model makes for easy portability. This allows for practice to occur in a variety of environments and promotes consistent use” (Langenfeld)  “The reusable task trainers are typically already present in any hospital or clinic that conducts simulation training and are all portable, allowing for easy transportation between training sites” (Kucera)  “E-learning overcomes this logistical difficulty by its ability to be accessed from anywhere with Internet access at a time convenient to the learner” (Bhatti)  “After the initial training session, a simulator would be given to each of them for independent use” (Langenfeld) |
| Minimising time away from clinical activities | “[E-learning packages] may be used to replace selected parts of a lecture-based timetable to ease pressure on the time of clinicians delivering the teaching without a loss in satisfaction or knowledge gained” (Bhatti)  “An asynchronous didactic component would be possible to decrease the time investment away from clinical activities, but the interactive nature of the session and the ability for learners to ask questions might decrease participant engagement” (Ginesi) |
| Transparent materials, transparent technique | “Transparent and flexible materials…may be better suited for the surgical training purposes” (Bangeas)  “The [model] glass is clear, which allows trainees or educators to watch their movements closely and make corrections as trainees transition from operating in open environments to confined environments” (Geary) |
| Replicating the operative challenges of the anorectal canal | “A three-ring binder was used to recreate angled visualization into the anal canal” (Langenfeld)  “Our model contributes to this body of literature highlighting creative use of materials to practice surgical skills in confined spaces” (Geary)  “Practicing colorectal surgeons scored the simulation as accurately recreating the visual, spatial and ergonomic challenges encountered in anorectal surgery practice” (Langenfeld) |
| To reinforce, not replace, clinical exposure | “The end goal would be increasing resident comfort with anorectal surgery, so they can maximally learn from and participate in anorectal surgery cases throughout their training” (Langenfeld)  “[The intervention can] address deficiencies in clinical exposure for general surgery residents” (Ginesi)  “[We are] developing a curriculum aimed at preparing medical students and residents of all levels for their colorectal rotations” (Langenfeld)  “Students and junior residents can use [the model] to gain initial familiarity with anorectal suturing, while more advanced learners can use it to hone their skills in more complicated tasks” (Langenfeld) |
| Uncertain translation to practice | “Future research will focus on demonstrating the effect of these trainers on operative performance” (Geary)  “It is important to assess whether gains form this workshop translate to improved diagnostic accuracy, technical abilities in the operating room, or test scores on the in-training examination” (Ginesi) |
| Focus on the fundamentals | “[Trainees] may require additional in vivo exposure to more complex procedures in order to gain confidence, they may benefit significantly from simple, cost-effective educational programs for basic anorectal skills” (Huang)  “All participants at all levels felt that the tasks were useful for practicing fundamental skills in confined spaces” (Geary) |
| Multispecialty input and output | “The website is regularly audited by…a range of medical professionals, nursing and managerial representation, as well as patient and community representatives” (Abdel-dayem)  “Owing to the high incidence of disease in patients treated by all specialties, formal benign anal disease curriculum needs to be introduced” (Kelley)  “This workshop could be extended to include other specialities, and may be valuable to physicians and trainees who frequently are the ‘first line’ for anorectal complaints” (Ginesi)  “Both surgical and medical specialities reported at 6 months that the material was applicable to their patient population, and overall improved their understanding of benign anal disease” (Kelley) |
| Diminishing returns with increasing experience | “[Educational benefits] are most pronounced for learners who have not had much previous exposure to anorectal diseases” (Langenfeld)  “Small numbers limited our ability to understand the training level in which these skills might plateau…further research is needed to better understand how this model discriminates between more discrete levels of training” (Geary)  “Suggestions for improvement were to stratify teaching based on resident level” (Ginesi)  “[Junior] residents improved their scores by the most and, therefore, will likely be the target population for future interventions” (Kelley) |
| *Note.* The first author of the study from which the quotation is derived is presented in brackets. | |

**Supplementary Table 1.** A selection of code labels and collated data extracts.
